# Formation of Biogenic Amines in *Pa* (Green Onion) Kimchi and *Gat* (Mustard Leaf) Kimchi

**DOI:** 10.3390/foods8030109

**Published:** 2019-03-24

**Authors:** Jun-Hee Lee, Young Hun Jin, Young Kyoung Park, Se Jin Yun, Jae-Hyung Mah

**Affiliations:** Department of Food and Biotechnology, Korea University, 2511 Sejong-ro, Sejong 30019, Korea; bory92@korea.ac.kr (J.-H.L.); younghoonjin3090@korea.ac.kr (Y.H.J.); eskimo@korea.ac.kr (Y.K.P.); ysj529@korea.ac.kr (S.J.Y.)

**Keywords:** kimchi, *Pa* kimchi, *Gat* kimchi, Korean specialty kimchi, biogenic amines, lactic acid bacteria, *Lactobacillus brevis*

## Abstract

In this study, biogenic amine content in *Pa* (green onion) kimchi and *Gat* (mustard leaf) kimchi, Korean specialty kimchi types, was determined by high-performance liquid chromatography (HPLC). Many kimchi samples contained low levels of biogenic amines, but some samples had histamine and tyramine content over the safe levels. Based on the comparative analysis between the ingredient information on food labels and biogenic amine content of kimchi samples, *Myeolchi*-*aekjeot* appeared to be an important source of biogenic amines in both kimchi. Besides, through the 16s rRNA sequence analysis, *Lactobacillus brevis* appeared to be responsible for the formation of biogenic amines (tyramine, β-phenylethylamine, putrescine, and cadaverine) in both kimchi, in a strain-dependent manner. During fermentation, a higher accumulation of tyramine, β-phenylethylamine, and putrescine was observed in both or one (for putrescine) of kimchi types when *L. brevis* strains served as inocula. The addition of *Myeolchi*-*aekjeot* affected the initial concentrations of most biogenic amines (except for spermidine in *Gat* kimchi) in both kimchi. Therefore, this study suggests that using appropriately salted and fermented seafood products for kimchi preparation and using biogenic amine-negative and/or biogenic amine-degrading starter cultures would be effective in reducing biogenic amine content in *Pa* kimchi and *Gat* kimchi.

## 1. Introduction

Biogenic amines (BA), including vasoactive amines (tryptamine, β-phenylethylamine, histamine, and tyramine), putrefactive amines (putrescine and cadaverine), and polyamines (spermidine and spermine), are present in a wide range of food products. Particularly, various types of fermented foods have been reported to contain BA, because these nitrogenous compounds are produced by microbial decarboxylation of amino acids [1]. While amine oxidases are able to detoxify most BA, excessive consumption of BA and/or amine oxidase inhibition by drugs, alcohol, and gastrointestinal disease have been reported to reduce the efficiency of the enzymes [1,2]. Consequently, unoxidized BA may cause symptoms such as diarrhea, hyperhidrosis, urticaria, hypotension, hypertension, headache, burning mouth, nausea, hot flush, respiratory distress, and cardiac arrest [2,3]. According to Silla Santos [3], the scombroid fish poisoning and cheese reaction are representative examples of foodborne illness associated with the intolerance to dietary histamine and tyramine, respectively. Thus, several studies have suggested the limits for consumption of BA as follows: β-phenylethylamine, 30 mg/kg; histamine, 100 mg/kg; tyramine, 100–800 mg/kg [4]; total BA, 1000 mg/kg [3].

According to the Codex standard [4], kimchi (in reality, *Baechu* kimchi) is defined as a salted and fermented food product made of Chinese cabbage (the main ingredient; *Baechu* in Korean) with a seasoning mixture consisting of red pepper powder, garlic, ginger, green onion, and radish. Other optional ingredients include fruits, glutinous rice paste, nuts, sugar, and salted and fermented seafood products [4]. Among various salted and fermented seafood products, such as *Jeotgal* (salted and fermented seafood) and *Aekjeot* (liquid part of *Jeotgal*), *Myeolchi-jeotgal* (salted and fermented anchovy), *Myeolchi-aekjeot* (salted and fermented anchovy sauce), and *Saeu-jeot* (salted and fermented shrimp) are the most popular condiments for the preparation of kimchi [5]. Meanwhile, kimchi has been used as a term standing for various types of lactic acid fermented vegetables in Korea. Nearly 190 types of kimchi have been developed based on regional styles and ingredients used [5,6]. Hence, people who live in eight provinces in Korea have differences in preferences for the types of kimchi, which include a number of kimchi varieties made of different main ingredients as well as *Baechu* kimchi [5]. For instance, people in North and South Jeolla provinces prefer *Pa* (green onion) kimchi and *Gat* (mustard leaf) kimchi, respectively, to *Baechu* kimchi. The main ingredients of *Pa* kimchi and *Gat* kimchi (the most popular regional specialty kimchi types) are green onion (*Allium wakegi* Araki) and mustard leaf (*Brassica juncea*), respectively, and have been reported to possess various health benefits, including antimicrobial and antioxidative effects [7,8,9,10]. Thus, the two types of regional specialty kimchi have gained increasing attention in Korea. In the meantime, Chang et al. [11] reported that kimchi varieties are sources for functional lactic acid bacteria (LAB) with probiotic activities; for example, *Lactobacillus acidophilus* with anti-neoplastic activity. Consequently, it has been expected (and scientifically proven) that kimchi varieties, including *Pa* kimchi and *Gat* kimchi, provide health benefits such as antimicrobial, antioxidative, antimutagenic, anticarcinogenic, anti-obesity, and anti-diabetic effects [6,12].

Differently from health benefits of LAB in kimchi, some species of LAB isolated from *Baechu* kimchi, *Kkakdugi*, and *Chonggak* kimchi have displayed tyramine production [13,14]. Since tyramine may lead to hypertensive crisis, headache, cerebral hemorrhage, and heart attack [15,16], the levels of BA, particularly tyramine, in kimchi varieties need to be investigated. While several studies have reported BA content in *Baechu* kimchi [17,18,19], only a single study has described BA content in *Pa* kimchi and *Gat* kimchi [17]. Therefore, the present study investigated bacterial production of BA as well as BA content in *Pa* kimchi and *Gat* kimchi. Fermentation of the two types of kimchi was also carried out to determine the predominant LAB species capable of producing BA throughout the fermentation period.

## 2. Materials and Methods

### 2.1. Samples Used

Fifteen samples of each type of kimchi (*Pa* kimchi and *Gat* kimchi) were purchased from retail markets, and stored at 4 °C until use. The broth of kimchi samples was subjected to analyses of BA as well as physicochemical and microbial properties. 

### 2.2. Measurements of Physicochemical Properties

Several physicochemical properties of *Pa* kimchi and *Gat* kimchi samples were measured as following. The pH was measured with a pH meter (Orion 3-star Benchtop, Thermo Scientific, Waltham, MA, USA). The salinity and titratable acidity were measured according to the Association of Official Analytical Chemists (AOAC) methods [20]. The water activity was measured by a water activity meter (AquaLab Pre, Meter Group, Inc., Pullman, WA, USA).

### 2.3. Measurements of Microbial Properties

Lactic acid bacterial count and total aerobic bacterial count were determined on de Man, Rogosa, and Sharpe (MRS) agar (Laboratorios Conda, Madrid, Spain) supplemented with Bromo-cresol purple (BCP; Samchun chemical, Ltd., Pyeongtaek, Korea) and Plate Count Agar (PCA; Difco, Becton Dickinson, Sparks, MD, USA), respectively. The incubation conditions were as follows: LAB, 37 °C for 48–72 h in an anaerobic chamber (Coy laboratory products Inc., Grass Lake, MI, USA) containing an atmosphere of 95% nitrogen and 5% hydrogen; total aerobic bacteria, 37 °C for 24–48 h in an incubator. After incubation, enumeration was carried out on plates with 30–300 colonies.

### 2.4. Isolation and Identification of LAB Strains

For the isolation of LAB strains, individual yellow colonies on MRS-BCP agar used for enumeration were picked and streaked on MRS agar, and incubated at 37 °C for 48–72 h. Single colonies were then streaked again on MRS agar, and incubated under the same condition to obtain pure cultures. The strains were then grown in MRS broth (Laboratorios Conda) under the same condition, and stored in the presence of 20% glycerol (*v*/*v*) at −80 °C.

For the identification of LAB strains, individual strains grown on MRS agar were subjected to sequence analysis of 16s rRNA gene amplified with the universal bacterial primer pair 518F and 805R (Solgent Co., Daejeon, Korea). The identities of sequences were determined using the Basic Local Alignment Search Tool (BLAST) of the National Center for Biotechnology Information (NCBI; http://www.ncbi.nlm.nih.gov/BLAST/).

### 2.5. Preparation and Fermentation of Pa Kimchi and Gat Kimchi

*Pa* kimchi and *Gat* kimchi were prepared according to the recipes described in previous studies [21,22] with minor modifications. Green onions or mustard leaves (the main ingredients of respective kimchi) were soaked in 10% (*w*/*v*) salt brine for 3 h. The salted main ingredients were rinsed with tap water three times, drained for 2 h, and mixed with a seasoning mixture consisting of red pepper powder, garlic, glutinous rice paste, and sugar. The desired values of salinity of *Pa* kimchi and *Gat* kimchi were 2.5%, respectively. The *Pa* kimchi and *Gat* kimchi samples were divided into five experimental groups, respectively, based on the presence or absence of *Myeolchi-aekjeot* (a condiment for kimchi) and LAB inoculum, as shown in Table 1. For this, three types of LAB strains for each kimchi, including a reference strain (*L. brevis* JCM1170 for both types of kimchi), two isolated strains capable of producing tyramine (*L. brevis*, PK3M04 for *Pa* kimchi, GK2M08 for *Gat* kimchi), and two isolated strains incapable of producing BA (*L. plantarum*, PK2M30 for *Pa* kimchi, GK2M12 for *Gat* kimchi), were inoculated onto the kimchi samples (all prepared with *Myeolchi-aekjeot*) at final concentrations of about 10^7^ CFU/g, if required. The isolated strains of *L. brevis* and *L. plantarum* served as inocula were originated from samples of the corresponding kimchi types, respectively. Two groups of non-inoculated kimchi were prepared with and without *Myeolchi-aekjeot*, respectively. Finally, the samples of five groups belonging to each type of kimchi were placed in polypropylene containers (25 × 15 × 20 cm), and *Pa* kimchi and *Gat* kimchi samples were fermented at 25 °C for 5 days and 25 °C for 3 days, respectively. The fermentation was carried out in duplicate.

### 2.6. BA Extraction from Kimchi Samples for HPLC Analysis

Analysis of BA in *Pa* kimchi and *Gat* kimchi samples was carried out based on the procedures developed by Eerola et al. [23] and modified by Ben-Gigirey et al. [24]. For BA extraction, 20 mL of 0.4 M perchloric acid (Sigma-Aldrich Chemical Co., St. Louis, MO, USA) were added to 5 g of kimchi broth, homogenized using a vortex mixer (Vortex-Genie, Scientific industries, Inc., Bohemia, NY, USA), and reacted in a cold chamber at 4 °C for 2 h. The mixture was centrifuged at 3000× *g* at 4 °C for 10 min, and the supernatant was collected. Subsequently, the residue was extracted again with an equal volume of 0.4 M perchloric acid under the same condition. Both supernatants were combined, and the final volume was adjusted to 50 mL with 0.4 M perchloric acid. The extract was filtered through Whatman paper No. 1 (Whatman International Ltd., Maidstone, UK).

### 2.7. BA Extraction from Lactic Acid Bacterial Cultures for HPLC Analysis

BA in lactic acid bacterial cultures were measured according to the procedures developed by Eerola et al. [23] and modified by Ben-Gigirey et al. [24,25]; the only exception was the culture medium used in this study. LAB strains were inoculated in 5 mL of MRS broth (pH 5.8) supplemented with 0.5% of L-histidine monohydrochloride monohydrate, L-tyrosine disodium salt hydrate, L-ornithine monohydrochloride, and L-lysine monohydrochloride, as well as with 0.0005% of pyridoxal-HCl (all from Sigma-Aldrich). After incubation at 37 °C for 48 h, 100 μL of the lactic acid bacterial broth culture was transferred to the same broth, and incubated under the same condition. Subsequently, the broth culture was filtered through a 0.2 μm membrane (Millipore Co., Bedford, MA, USA). Nine milliliters of 0.4 M perchloric acid were then added to 1 mL of the filtered broth culture, and mixed using a vortex mixer. The mixture was reacted in a cold chamber at 4 °C for 2 h, and centrifuged at 3000× *g* at 4 °C for 10 min. The supernatant (viz., the BA extract from LAB culture) was filtered through Whatman paper No. 1.

### 2.8. Preparation of Standard Solutions for HPLC Analysis

Stock standard solutions of BA, including tryptamine, β-phenylethylamine hydrochloride, putrescine dihydrochloride, cadaverine dihydrochloride, histamine dihydrochloride, tyramine hydrochloride, spermidine trihydrochloride, and spermine tetrahydrochloride (all from Sigma-Aldrich), were separately prepared at 10,000 mg/L concentration in deionized water. Working solutions at 1000 mg/L concentration were prepared by diluting 1 mL of each stock solution in deionized water to bring to a final volume of 10 mL. The concentrations of all standard solutions used were 0, 10, 50, 100, and 1000 mg/L. In addition, 1,7-diaminoheptane (Sigma-Aldrich) served as an internal standard.

### 2.9. Derivatization of Extracts and Standards

Derivatization of BA was carried out according to the procedures developed by Ben-Gigirey et al. [24]. One milliliter of extract (or standard solution) prepared above was mixed with 200 μL of 2 M sodium hydroxide and 300 μL of saturated sodium bicarbonate (all from Sigma-Aldrich). Two milliliters of a dansyl chloride (Sigma-Aldrich) solution (10 mg/mL) prepared in acetone were added to the mixture, and reacted at 40 °C for 45 min. Residual dansyl chloride was removed by adding 100 μL of 25% ammonium hydroxide (Sigma-Aldrich). After reaction at 25 °C for 30 min, the final volume was adjusted to 5 mL with acetonitrile. Finally, the mixture was centrifuged at 3000× *g* for 5 min, and the supernatant was filtered through a 0.2 μm-pore-size filter (Millipore). The filtered supernatant was kept at −25 °C until assayed by HPLC.

### 2.10. Chromatographic Separations

Chromatographic separation of BA was carried out according to the procedures developed by Ben-Gigirey et al. [24] with minor modifications. An HPLC unit (YL9100, YL Instruments Co., Ltd., Anyang, Korea) equipped with a UV-vis detector (YL Instruments) and with Autochro-3000 data system (YL Instruments) was used. A Nova-Pak C_18_ 4 μm column (150 mm × 4.6 mm, Waters, Milford, MA, USA) held at 40 °C was used with 0.1 M ammonium acetate (solvent A; Sigma-Aldrich) and acetonitrile (solvent B; SK chemicals, Ulsan, Korea) as the mobile phases adjusted to the flow rate of 1 mL/min. The program was set for a linear gradient starting from 50% of solvent B to reach 90% of the solvent at 19 min. The injection volume was 10 μL, and monitored at 254 nm. The limits of detection were approximately 0.1 μg/mL for all BA in standard solutions and in lactic acid bacterial cultures, and about 0.1 mg/kg for BA in food matrices. The procedure for BA analysis, from extraction to HPLC analysis, was illustrated in our previous article [14].

### 2.11. Statistical Analyses

The data were presented as means and standard deviations of triplicate experiments. Statistical outliers in data were eliminated according to the Grubbs outlier test (α = 0.05). The significance of differences was determined by one-way analysis of variance (ANOVA) with Fisher’s multiple comparison module of the Minitab statistical software, version 17.1 (Minitab Inc., State College, PA, USA). Differences with *p* values of <0.05 were considered statistically significant. Differences with *p* values of <0.05 were considered statistically significant.

## 3. Results and Discussion

### 3.1. BA Content in Pa Kimchi and Gat Kimchi

As shown in Table 2, concentrations of BA in commercial products of *Pa* kimchi and *Gat* kimchi were measured to determine whether the amounts of BA in both kimchi are within the safe levels for consumption based on the suggestions of Ten Brink et al. [1] and Silla Santos [3]. Among the data obtained from 15 samples of each type of kimchi, statistical outliers in datasets generated from two samples of each kimchi type were respectively excluded from the results, and thereby data from 13 samples of each kimchi type were used for calculation and interpretation of the experimental results. The ranges (minimum to maximum) of BA content measured in *Pa* kimchi were as follows: Tryptamine, not detected (ND)–15.95 mg/kg; β-phenylethylamine, ND–5.97 mg/kg; putrescine, ND–254.47 mg/kg; cadaverine, ND–123.29 mg/kg; histamine, 8.67–386.03 mg/kg; tyramine, ND–181.10 mg/kg; spermidine, 2.32–18.74 mg/kg; spermine, ND–33.84 mg/kg (the upper part of Table 2). In *Gat* kimchi samples, the ranges of BA in the same order as above were ND–26.74 mg/kg, ND–15.75 mg/kg, 1.89–720.82 mg/kg, 2.12–52.43 mg/kg, 3.30–232.10 mg/kg, 1.28–142.06 mg/kg, 12.26–32.62 mg/kg, and ND–61.94 mg/kg, respectively (the lower part of Table 2). In agreement with the results, a wide range of BA content in *Baechu* kimchi has been reported previously [18,19]. On the other hand, Mah et al. [17] reported the low levels (below 30 mg/kg) of putrescine, cadaverine, histamine, tyramine, spermidine, and spermine in both *Pa* kimchi and *Gat* kimchi, all showing narrow ranges of BA content. Somewhat disparate observations between the present and previous studies might result from manufacturing methods, ingredients, and storage condition of kimchi samples [26].

In the present study, many samples of *Pa* kimchi and *Gat* kimchi had low levels of β-phenylethylamine (below 30 mg/kg) and other BA (below 100 mg/kg), which were all within the safe levels for consumption. However, 6 of 13 (46%) *Pa* kimchi and 2 of 13 (15%) *Gat* kimchi samples contained about two to four times higher levels of histamine than the safe level of the amine (100 mg/kg) suggested by Ten Brink et al. [1]. Meanwhile, 5 of 13 (38%) *Pa* kimchi and 6 of 13 (46%) *Gat* kimchi samples had approximately one to two times higher levels of tyramine compared to the safe level of the amine (100 mg/kg) suggested by Ten Brink et al. [1]. As for the levels of putrefactive amines, 4 of 13 (31%) *Pa* kimchi and 3 of 13 (23%) *Gat* kimchi samples contained putrescine over 100 mg/kg, and 2 of 13 (15%) *Pa* kimchi samples had over 100 mg/kg of cadaverine. Particularly, one (GK23) of the *Gat* kimchi samples contained 720.82 ± 37.04 mg/kg (mean ± standard deviation) of putrescine, so that total BA content was over the safe level of 1000 mg/kg suggested by Silla Santos [3]. Taken together, although many samples of *Pa* kimchi and *Gat* kimchi seem to be safe for consumption, proper monitoring and reduction of BA in both types of kimchi are required to reduce the risk of ingesting high levels of BA due to the presence of not only high levels of histamine and tyramine in several samples, but also a significant level of total BA, exceeding the safe levels. This is in disagreement with a previous report in which low levels of BA were detected in *Pa* kimchi and *Gat* kimchi as described above [17]. Different findings between the present and previous studies imply that the BA content in both types of kimchi can be reduced. 

Though the main ingredients in kimchi varieties are vegetables, salted and fermented seafood products such as *Jeotgal* and *Aekjeot* are commonly used as condiments for the preparation of kimchi. Among the salted and fermented seafood products, *Myeolchi*-*aekjeot* is one of the most widely used condiments for kimchi preparation and makes up approximately 8%–15% of *Pa* kimchi (on the basis of weight percent) and 5%–9% of *Gat* kimchi, respectively [7,21,22,27]. Likely, most samples used in the present study were prepared with *Myeolchi-aekjeot*, according to food labels, although the labels just provided the list of ingredients (but not the content). In previous studies, Mah et al. [17] and Cho et al. [18] reported that *Myeolchi-aekjeot* contained significantly high levels of histamine, tyramine, putrescine, and cadaverine. Thus, high levels of these four BA in several samples of *Pa* kimchi and *Gat* kimchi in this study might be, at least partially, originated from *Myeolchi-aekjeot*. This speculation is in part supported by a previous report by Kang [19] that histamine content was higher in *Baechu* kimchi prepared with *Myeolchi-aekjeot* (592.78 ± 3.43 mg/kg) than in that without *Myeolchi-aekjeot* (77.13 ± 0.39 mg/kg). Therefore, when preparing *Pa* kimchi and *Gat* kimchi, condiment adjustments (such as using *Myeolchi-aekjeot* with low BA content, reducing the amount of *Myeolchi-aekjeot* used, and/or replacing *Myeolchi-aekjeot* with other salted and fermented seafood products) would be helpful in reducing BA content in the kimchi varieties.

### 3.2. Physicochemical and Microbial Properties of *Pa* Kimchi and *Gat* Kimchi

The values of pH, salinity, titratable acidity, water activity, total aerobic bacterial count, and lactic acid bacterial count in *Pa* kimchi and *Gat* kimchi were measured to see whether the properties are related to BA content. The properties measured in *Pa* kimchi were as follows: pH, 4.26–5.70 (5.19 ± 0.37, mean ± standard deviation); salinity, 1.28–2.73% (2.10 ± 0.40%); titratable acidity, 0.41–1.28% (0.68 ± 0.27%); water activity_,_ 0.980–0.990 (0.990 ± 0.010); total aerobic bacterial count, 6.41–9.04 Log CFU/mL (7.43 ± 0.78 Log CFU/mL); lactic acid bacterial count, 5.78–8.74 Log CFU/mL (7.04 ± 0.84 Log CFU/mL). In *Gat* kimchi, the properties (in the same order as above) were as follows: pH, 4.26–5.30 (4.57 ± 0.37); salinity, 1.34–2.35% (1.96 ± 0.34%); titratable acidity, 0.53–1.23% (0.81 ± 0.26%); water activity_,_ 0.973–0.989 (0.980 ± 0.010); total aerobic bacterial count, 6.37–8.11 Log CFU/mL (7.39 ± 0.65 Log CFU/mL); lactic acid bacterial count, 6.37–8.21 Log CFU/mL (7.18 ± 0.80 Log CFU/mL). The values are in accordance with those of previous reports on *Pa* kimchi and *Gat* kimchi [7,21]. Silla Santos [3] and Lu et al. [28] suggested that certain physicochemical and microbial properties are related to BA formation in fermented foods. In this study, linear regression analyses were carried out between the values of the properties and BA content. However, the content of respective BA had weak correlations with the respective properties in this study (data not shown). Therefore, the BA content detected in *Pa* kimchi and *Gat* kimchi might be affected by complex combinations of more than one factors (or/as multiple variable), including the addition of *Myeolchi*-*aekjeot*.

### 3.3. BA Production by LAB Strains Isolated from *Pa* Kimchi and *Gat* Kimchi

A total of 202 LAB strains (99 and 103 strains from *Pa* kimchi and *Gat* kimchi, respectively) were tested for in vitro BA production. Most LAB strains produced low levels of BA (below the detection limit). However, 16 of 99 LAB strains from *Pa* kimchi displayed three different patterns of BA production: (i) Strong production of tyramine along with β-phenylethylamine (seven strains), (ii) strong production of putrescine and cadaverine (eight strains), and (iii) simultaneous production of tyramine, β-phenylethylamine, putrescine, and cadaverine (one strain). Of the 103 LAB strains from *Gat* kimchi, 11 LAB strains produced BA with two different patterns, i.e., 9 LAB strains produced BA following pattern (i), and 2 LAB strains followed pattern (ii). Considering the high levels of histamine, tyramine, putrescine, and cadaverine measured in several samples of *Pa* kimchi and *Gat* kimchi (see Section 3.1), the LAB strains capable of producing tyramine, putrescine, and cadaverine might significantly contribute to the content of, at least, these three BA in both kimchi. On the other hand, histamine production was not detected from all the LAB strains. Thus, histamine content in both kimchi appeared to be attributed to the *Myeolchi*-*aekjeot* rather than LAB strains as described in Section 3.1.

To determine LAB species responsible for BA production in *Pa* kimchi and *Gat* kimchi, a total of 27 BA-producing LAB strains were identified based on 16s rRNA sequence analysis, as shown in Table 3. The LAB strains were identified as *L. brevis* (21 strains), *L. sakei* (2 strains), *Enterococcus faecium* (2 strains), and *Leuconostoc mesenteroides* (2 strains). Of the *L. brevis* strains, 10 strains produced 1.27–2.39 μg/mL of β-phenylethylamine and 278.57–365.96 μg/mL of tyramine, whereas another 10 strains produced 313.43–322.21 μg/mL of putrescine and 39.24–53.06 μg/mL of cadaverine (all 20 strains did not produce other BA). The other one strain simultaneously produced β-phenylethylamine, tyramine, putrescine, and cadaverine, exhibiting a somewhat weaker production of the former two BA and a little stronger production of the latter two BA. In accordance with the results, *L. brevis* has displayed a strain-dependent (as well as species-dependent) capacity to produce BA in previous studies [29,30]. Meanwhile, BA production by the rest six LAB strains, producing only β-phenylethylamine and tyramine, were as follows: *L. sakei,* 1.00 and 3.96 μg/mL of β-phenylethylamine, and 113.98 and 131.36 μg/mL of tyramine; *E. faecium*, 3.51 and 3.88 μg/mL of β-phenylethylamine, and 259.10 and 269.57 μg/mL of tyramine; *Leu. mesenteroides*, 1.47 and 1.91 μg/mL of β-phenylethylamine, and 145.14 and 301.67 μg/mL of tyramine. Similarly, the production of β-phenylethylamine and tyramine by *L. sakei*, *Leu. mesenteroides*, and *E. faecium* has been reported in previous studies [13,29,31]. Nevertheless, *L. brevis* is most likely responsible for BA formation in *Pa* kimchi and *Gat* kimchi, because the species was not only more abundant, but also revealed a larger production of most BA (except for β-phenylethylamine production by *E. faecium*) than other species in this study. In addition, all of the tyramine-producing LAB strains simultaneously produced a trace amount of β-phenylethylamine. This might be because bacterial tyrosine decarboxylase produces not only tyramine from tyrosine, but also β-phenylethylamine from phenylalanine [30,32].

For the 175 LAB strains incapable of producing BA (below the detection limit), about 30 strains were randomly selected, and subsequently identified as *L. brevis* (3 strains), *L. plantarum* (8 strains), *Leu. mesenteroides* (18 strains), and *Pediococcus pentosaceus* (1 strain). All the species have been reported as dominant species in previous studies on *Baechu* kimchi [6,13]. Thus, all the strains seem to have potential as BA-negative starter cultures for kimchi fermentation if they fulfill the criteria of starter culture [33]. Interestingly, it appeared in this study that, while certain strains of *L. brevis* and *Leu. mesenteroides* could produce high levels of BA, other strains belonging to the same species could not, which suggests that the ability of LAB strains to produce BA is determined at strain level. Therefore, although a bacterial species has been commonly considered to produce BA, some strains of this species may be able to serve as BA-negative and/or BA-degrading starter cultures depending on their abilities [34].

### 3.4. Changes in BA Content during Fermentation of Pa Kimchi and Gat Kimchi

As shown in Table 1, five experimental groups of *Pa* kimchi and *Gat* kimchi were prepared with/without *Myeolchi*-*aekjeot* (a condiment used for kimchi preparation) and with/without selected LAB strains (*L. brevis* and *L. plantarum* as inocula) to investigate the contributions of the condiment and inoculum to BA content during the fermentation of both types of kimchi. *L. brevis* strains of PK3M04 (for *Pa* kimchi) and GK2M08 (for *Gat* kimchi) with the highest tyramine production capabilities among the isolated LAB strains were employed as inocula to investigate whether the strains of *L. brevis* practically contribute to BA formation (particularly tyramine) in *Pa* kimchi and *Gat* kimchi (refer to Section 3.3). On the other hand, *L. plantarum* strains of PK2M30 (for *Pa* kimchi) and GK2M12 (for *Gat* kimchi) were used as counterparts of *L. brevis* strains because the strains were unable to produce BA in this study, which is also supported by previous reports that *L. plantarum* was incapable of producing BA [29,30].

**Figure 1:**
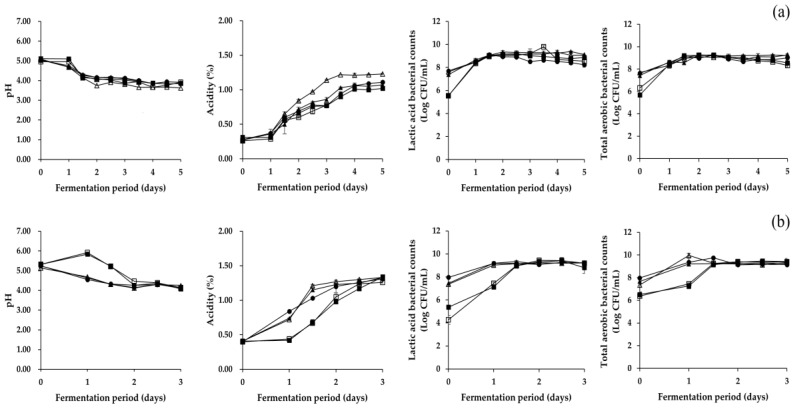
Changes in physicochemical and microbial properties during fermentation of (**a**) *Pa* kimchi and (**b**) *Gat* kimchi. □: B (no addition of *Myeolchi*-*aekjeot*, no inoculum), ■: C (addition of *Myeolchi*-*aekjeot*, no inoculum), ●: R (addition of *Myeolchi*-*aekjeot*, *L. brevis* JCM1170 for both kimchi), ▲: LB (addition of *Myeolchi*-*aekjeot*, *L. brevis* PK3M04 for *Pa* kimchi, *L. brevis* GK2M08 for *Gat* kimchi), △: LP (addition of *Myeolchi*-*aekjeot*, *L. plantarum* PK2M30 for *Pa* kimchi, *L. plantarum* GK2M12 for *Gat* kimchi).

As presented in Figure 1, changes in physicochemical and microbial properties of *Pa* kimchi and *Gat* kimchi were similar with those of previous studies [7,21]. In detail, *Pa* kimchi groups displayed a slower acidification than *Gat* kimchi groups. Similarly, Lee et al. [21] reported a slower fermentation progress in *Pa* kimchi than in *Baechu* kimchi. Thus, fermentation of *Pa* kimchi and *Gat* kimchi was carried out for five days and three days, respectively. Changes in both pH and titratable acidity measured in the LP group of *Pa* kimchi were slightly faster than those in the other groups of the kimchi, and the values of all the groups remained constant in the later period of fermentation. In *Gat* kimchi, the pH and titratable acidity measured in the groups inoculated with LAB strains (LB, LP, and R groups) were altered much faster than non-inoculated groups (B and C groups), and the values remained constant in the later period of fermentation. Meanwhile, all groups of *Pa* kimchi and *Gat* kimchi exhibited an increment in microbial counts (either total aerobic bacteria or lactic acid bacteria) during the fermentation for 1.5 days, and remained constant thereafter. The salinity of *Pa* kimchi and *Gat* kimchi was altered from 2.37 ± 0.06 to 2.07 ± 0.04, and from 3.03 ± 0.16 to 2.79 ± 0.06, respectively, showing a slight decline in the value (data not shown). The steady reduction of salinity might be due to osmosis between the main ingredient (either green onion or mustard leaf) and kimchi broth (containing seasoning mixture), as suggested by Shin et al. [35]. The water activity of *Pa* kimchi and *Gat* kimchi remained constant at 0.970 ± 0.002 and 0.959 ± 0.004, respectively, throughout the fermentation period (data not shown).

**Figure 2:**
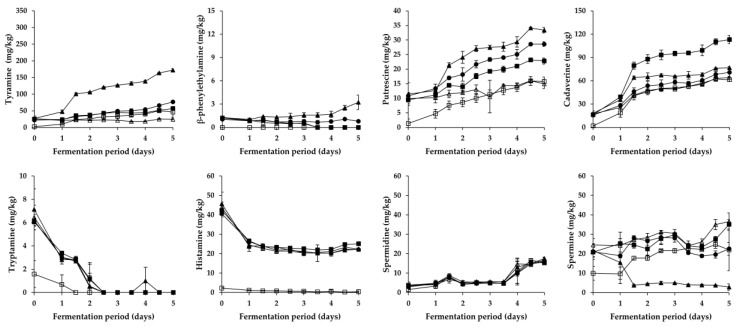
Changes in BA content in *Pa* kimchi during fermentation. □: B (no addition of *Myeolchi*-*aekjeot*, no inoculum), ■: C (addition of *Myeolchi*-*aekjeot*, no inoculum), ●: R (addition of *Myeolchi*-*aekjeot*, *L. brevis* JCM1170), ▲: LB (addition of *Myeolchi*-*aekjeot*, *L. brevis* PK3M04), △: LP (addition of *Myeolchi*-*aekjeot*, *L. plantarum* PK2M30).

**Figure 3:**
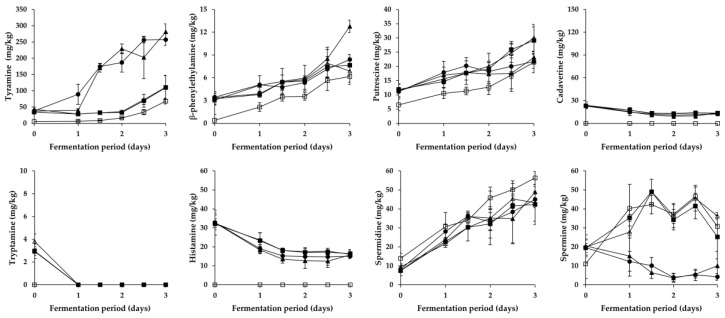
Changes in BA content in *Gat* kimchi during fermentation. □: B (no addition of *Myeolchi*-*aekjeot*, no inoculum), ■: C (addition of *Myeolchi*-*aekjeot*, no inoculum), ●: R (addition of *Myeolchi*-*aekjeot*, *L. brevis* JCM1170), ▲: LB (addition of *Myeolchi*-*aekjeot*, *L. brevis* GK2M08), △: LP (addition of *Myeolchi*-*aekjeot*, *L. plantarum* GK2M12).

As shown in Figure 2 and Figure 3, overall changes in the content of respective BA during the fermentation of *Pa* kimchi and *Gat* kimchi were similar with those of a previous report on *Kkakdugi* and *Chonggak* kimchi [14]. In detail, tyramine content steadily increased in most groups of both kimchi, except for the LP group of *Pa* kimchi. The increment might be caused by either inoculated or indigenous tyramine-producing LAB strains (most likely *L. brevis*; refer to Section 3.3). Thus, R and LB groups of both kimchi that had been inoculated with tyramine-producing *L. brevis* strains revealed a larger increment of tyramine content than the other groups. In contrast, the tyramine content in LP groups either remained constant (in *Pa* kimchi) or increased relatively slightly (in *Gat* kimchi) because *L. plantarum* inocula were selected due to their incapability of BA production [29,30]. The levels of tyramine in B and C groups of both kimchi were much lower than those in R and LB groups, but either slightly higher than that in LP group of *Pa* kimchi or slightly lower than that in LP group of *Gat* kimchi. Thus, tyramine in the B and C groups might be formed by indigenous tyramine-producing LAB strains other than *L. plantarum*. Moreover, since tyramine content was higher in C group than in B group, bacterial strains derived from *Myeolchi*-*aekjeot* might also affect the content. In addition, β-phenylethylamine in all groups exhibited almost the same patterns of alterations as tyramine in the groups throughout the fermentation of both kimchi, as expected based on previous reports [30,32], although the content was much lower than tyramine.

Differently from tyramine and β-phenylethylamine, tryptamine content in all groups of both kimchi steadily decreased during the earlier period of fermentation. Among the groups, B group (prepared without *Myeolchi*-*aekjeot*) had the lowest initial concentration, regardless of the types of kimchi. Likely, the lowest histamine content was detected in the B groups of both kimchi. Thus, the initial concentrations of tryptamine and histamine seem to come from *Myeolchi*-*aekjeot*. This is in agreement with previous studies suggesting that *Myeolchi*-*aekjeot* is a source of histamine in kimchi [14,19]. In addition to the tryptamine and histamine, similar differences in the content of other BA were also observed between B group and the other groups of both types of kimchi (except for spermidine in *Gat* kimchi) in this study. Therefore, it turned out that *Myeolchi*-*aekjeot* serves as an important source of not only histamine, but also other BA in kimchi. Supporting this assumption, as mentioned in Section 3.1, significantly high levels of BA, including tryptamine (<296.8 mg/kg), β-phenylethylamine (<54.1 mg/kg), putrescine (<182.1 mg/kg), cadaverine (<263.6 mg/kg), histamine (<1154.7 mg/kg), tyramine (<611.3 mg/kg), spermidine (<358.6 mg/kg), and spermine (<12.2 mg/kg), have been previously reported in *Myeolchi-aekjeot* [17,18]. Meanwhile, in most groups (except for B group), histamine content significantly decreased during the earlier period of fermentation, and remained constant in the later period, which is in accordance with previous reports by Kim et al. [36] and Jin et al. [14] that inoculated and indigenous LAB strains degraded histamine during the fermentation period.

As for putrefactive amines, putrescine content in all groups of *Pa* kimchi and *Gat* kimchi steadily increased throughout the fermentation period, as reported in previous studies on *Baechu* kimchi, *Kkakdugi*, and *Chonggak* kimchi [14,17]. In *Pa* kimchi, the groups inoculated with *L. brevis* (LB and R groups in order of amount) contained higher levels of putrescine than C and LP groups (in the same order), which indicates that while *L. brevis* significantly produced putrescine, *L. plantarum* likely degraded the putrescine because the lowest putrescine level was observed in LP group. In contrast, in the case of *Gat* kimchi, the levels of putrescine in C and LP groups were higher than those in LB and R groups. Thus, it seems that the *L. brevis* and *L. plantarum* work differently depending upon the types of kimchi. In the meantime, cadaverine content in *Pa* kimchi was the highest in C group, and followed by the groups inoculated with LAB strains (LB, R, and LP groups). This suggests that in *Pa* kimchi, bacterial strains originated from *Myeolchi*-*aekjeot* might influence the cadaverine content, and LAB strains could somehow (most likely their antimicrobial action) inhibit the cadaverine production by those bacterial strains from *Myeolchi*-*aekjeot*. In addition, cadaverine content in all groups constantly increased during the fermentation period of *Pa* kimchi. On the contrary, the cadaverine content in all groups of *Gat* kimchi slightly decreased throughout the fermentation period. Taken together, *Pa* kimchi and *Gat* kimchi displayed different patterns of putrefactive amine production (or degradation), which might be attributed to complex factors (and combinations thereof), including distinct indigenous strains as well as physicochemical properties resulting from different ingredients (especially main ingredients). Further research is required to clarify this issue.

Spermidine content in all groups of *Pa* kimchi and *Gat* kimchi steadily increased throughout the fermentation period, which is in agreement with observations in a previous study in which the spermidine content increased during the fermentation of *Kkakdugi* and *Chonggak* kimchi [14]. Particularly, *Pa* kimchi showed a much smaller increment of spermidine content than *Gat* kimchi. *Pa* kimchi also exhibited a statistically insignificant difference in spermidine content among all the groups on each day of the fermentation, while B group of *Gat* kimchi contained a statistically higher level of spermidine than C, R, and LP groups in the later period of fermentation. As described above, B groups of both kimchi showed lower BA levels than the other groups. However, the spermidine content in the B group of *Gat* kimchi is one exception to this rule, and the reason for this is unclear. Aside from spermidine, the content of spermine (another polyamine) in LB group of *Pa* kimchi and in LB and R groups of *Gat* kimchi revealed a decrement during the earlier period of fermentation, which indicates that *L. brevis* could degrade this polyamine. Similarly, there is a report of spermine-degrading *L. brevis* isolated from a traditional Italian cheese [37]. In the meantime, spermine content in the other groups of both kimchi in this study gradually increased during the earlier period of fermentation, and then decreased during the later period (except for C and LP groups of *Pa* kimchi showing a continuous increment). In both types of kimchi, interestingly, the levels of tyramine and β-phenylethylamine were higher in the LB and R groups inoculated with *L. brevis* than in the other groups, whereas an almost complete reversed order of the groups was found when spermine content was compared. Similar patterns were also observed in a previous study [14]. Thus, spermine content seems to be negatively related to microbial production of tyramine and β-phenylethylamine. Further research is required to make it clear. Together with the observations described in Section 3.1 and Section 3.3, it turned out that *Myeolchi*-*aekjeot* and LAB strains influence either the formation or the degradation of BA in several ways during the fermentation of *Pa* kimchi and *Gat* kimchi.

## 4. Conclusions

This study indicated that the amounts of BA in many samples of *Pa* kimchi and *Gat* kimchi were within the safe levels for human consumption, but several samples contained histamine and tyramine over the safe levels of the respective BA (100 mg/kg for both BA, respectively). It was also found that while *Myeolchi*-*aekjeot* was an important source of BA in both *Pa* kimchi and *Gat* kimchi, *L. brevis* strains from both types of kimchi had a strain-dependent capacity to produce both β-phenylethylamine and tyramine, and/or both putrescine and cadaverine. The physicochemical and microbial properties of both types of kimchi exhibited weak correlations with BA content in the corresponding kimchi types in the present study. Through the fermentation of *Pa* kimchi and *Gat* kimchi, it turned out that *L. brevis* is responsible for the formation of BA, including β-phenylethylamine, tyramine, and putrescine, in both types of kimchi, and *Myeolchi*-*aekjeot* significantly affects the BA content in both *Pa* kimchi and *Gat* kimchi, except for spermidine in *Gat* kimchi.

Interestingly, the groups inoculated with *L. brevis* strains contained higher levels of tyramine and β-phenylethylamine, but much lower levels of spermine (probably due to degradation by the strains) than the other groups throughout the fermentation of *Pa* kimchi and *Gat* kimchi. Also, inoculated and indigenous LAB strains significantly degraded both tryptamine and histamine during the fermentation of both types of kimchi. The results imply that the LAB strains, including *L. brevis*, may have a potential to degrade specific BA in kimchi. In addition, differences in the production patterns of putrescine, cadaverine, and spermidine were observed between *Pa* kimchi and *Gat* kimchi. This might be because distinct complex factors (and their combinations) present in the respective types of kimchi differently influence the BA production in the kimchi varieties, which may need to be further studied.

Taken together, this study suggests two important measures for reducing BA in *Pa* kimchi and *Gat* kimchi, as follows: (i) The alteration of the ratio of *Myeolchi*-*aekjeot* to other ingredients used for kimchi preparation, and (ii) use of starter cultures other than BA-producing *L. brevis* strains, particularly BA-negative and/or -degrading LAB starter cultures.

## Figures and Tables

**Table 1:** Ingredients used for preparation of *Pa* (green onion) kimchi and *Gat* (mustard leaf) kimchi.

Ingredients (g)	Experimental Groups				
	B (Blank group)	C (Control group)	R (Reference group)	LB (*L. brevis* group)	LP (*L. plantarum* group)
Main Ingredient ^1^	1500	1500	1500	1500	1500
Red Pepper Powder	120	120	120	120	120
Garlic	30	30	30	30	30
Ginger	30	30	30	30	30
Glutinous Rice Paste ^2^	225	225	225	225	225
Sugar	30	30	30	30	30
*Myeolchi*-*aekjeot*	-	150	150	150	150
*L. brevis* JCM1170	-	-	10^7^ CFU/g	-	-
*L. brevis* ^3^	-	-	-	10^7^ CFU/g	-
*L. plantarum* ^4^	-	-	-	-	10^7^ CFU/g

^1^ Salted green onion or salted mustard leaf; ^2^ glutinous rice flour:water = 1:10; ^3^ *L. brevis* PK3M04 for *Pa* kimchi, *L. brevis* GK2M08 for *Gat* kimchi; ^4^ *L. plantarum* PK2M30 for *Pa* kimchi, GK2M12 for *Gat* kimchi.

**Table 2:** Biogenic amines (BA) content in *Pa* (green onion) kimchi and *Gat* (mustard leaf) kimchi samples.

Samples ^1^	BA Content (mg/kg) ^2^							
	TRP	PHE	PUT	CAD	HIS	TYR	SPD	SPM
PK11	14.92 ± 3.35 ^A,3^	3.39 ± 0.01 ^C^	254.47 ± 16.83 ^A^	63.43 ± 5.77 ^C^	286.04 ± 26.18 ^BC^	150.81 ± 11.90 ^B^	12.87 ± 1.72 ^C^	27.90 ± 9.08 ^ABC^
PK12	11.56 ± 1.20 ^BC^	4.65 ± 0.94 ^B^	158.33 ± 21.71 ^B^	53.83 ± 7.36 ^CD^	318.67 ± 19.21 ^B^	181.10 ± 18.00 ^A^	13.01 ± 1.46 ^BC^	24.37 ± 1.72 ^ABCD^
PK13	15.95 ± 3.91 ^A^	5.97 ± 1.20 ^A^	94.99 ± 5.91 ^D^	82.73 ± 8.38 ^B^	386.03 ± 33.13 ^A^	179.98 ± 14.26 ^A^	12.21 ± 1.98 ^C^	25.87 ± 2.72 ^ABCD^
PK14	6.33 ± 0.42 ^DE^	2.34 ± 0.15 ^D^	137.71 ± 12.78 ^C^	38.56 ± 6.53 ^DE^	249.07 ± 9.01 ^C^	141.12 ± 2.10 ^BC^	7.15 ± 3.63 ^DE^	26.97 ± 7.00 ^ABC^
PK15	10.26 ± 0.73 ^C^	1.81 ± 0.23 ^D^	135.55 ± 0.64 ^C^	36.83 ± 0.14 ^DE^	226.72 ± 0.39 ^C^	127.59 ± 3.98 ^C^	5.75 ± 2.05 ^EF^	ND ^F,4^
PK16	13.74 ± 3.13 ^AB^	3.28 ± 0.25 ^C^	69.98 ± 1.74 ^E^	119.68 ± 32.16 ^A^	300.73 ± 59.36 ^B^	ND ^D^	16.55 ± 1.92 ^AB^	10.40 ± 1.84 ^E^
PK21	5.20 ± 1.37 ^DE^	ND ^E^	6.05 ± 0.25 ^G^	11.02 ± 2.12 ^F^	62.60 ± 2.55 ^D^	16.31 ± 2.62 ^D^	7.79 ± 1.49 ^DE^	24.88 ± 1.74 ^ABCD^
PK31	ND ^G^	ND ^E^	18.65 ± 1.81 ^FG^	123.29 ± 8.97 ^A^	45.72 ± 4.51 ^DEF^	5.53 ± 0.86 ^D^	12.40 ± 1.36 ^C^	22.02 ± 4.24 ^BCD^
PK41	3.30 ± 0.59 ^EF^	ND ^E^	7.56 ± 0.22 ^G^	ND ^F^	23.45 ± 0.18 ^EFG^	15.99 ± 2.12 ^D^	4.72 ± 1.15 ^EF^	17.81 ± 2.68 ^CDE^
PK42	1.12 ± 1.28 ^FG^	ND ^E^	ND ^G^	ND ^F^	8.67 ± 9.91 ^G^	7.50 ± 8.72 ^D^	2.32 ± 2.95 ^F^	16.07 ± 14.20 ^DE^
PK43	1.96 ± 0.53 ^FG^	ND ^E^	6.43 ± 0.86 ^G^	2.52 ± 0.92 ^F^	14.72 ± 0.05 ^FG^	17.65 ± 3.20 ^D^	5.34 ± 3.10 ^EF^	33.84 ± 6.70 ^A^
PK51	6.48 ± 0.96 ^D^	ND ^E^	31.47 ± 9.56 ^F^	10.91 ± 1.33 ^F^	53.12 ± 2.35 ^DE^	20.30 ± 1.49 ^D^	18.74 ± 2.77 ^A^	29.71 ± 8.02 ^AB^
PK52	ND ^G^	1.57 ± 0.64 ^D^	24.34 ± 1.87 ^F^	30.08 ± 3.49 ^E^	50.57 ± 3.90 ^DE^	5.58 ± 0.73 ^D^	10.03 ± 0.78 ^CD^	22.86 ± 1.10 ^BCD^
Average	6.99 ± 5.74	1.77 ± 2.04	78.79 ± 79.00	44.07 ± 42.85	155.85 ± 139.26	66.88 ± 74.91	9.91 ± 4.89	21.75 ± 8.94
GK11	4.86 ± 0.15 ^DE^	ND ^E^	9.38 ± 1.19 ^HI^	8.61 ± 0.90 ^CD^	11.27 ± 1.13 ^C^	9.99 ± 0.05 ^GH^	13.01 ± 0.36 ^FG^	24.83 ± 7.96 ^DE^
GK21	19.53 ± 6.88 ^AB^	ND ^E^	25.35 ± 1.52 ^GH^	9.90 ± 0.96 ^CD^	30.81 ± 0.86 ^C^	138.45 ± 3.83 ^B^	12.26 ± 5.86 ^G^	ND ^F^
GK22	18.06 ± 5.31 ^BC^	ND ^E^	36.52 ± 1.68 ^FG^	16.18 ± 1.47 ^C^	39.08 ± 1.76 ^BC^	142.06 ± 7.71 ^A^	13.07 ± 4.65 ^EFG^	ND ^F^
GK23	26.74 ± 0.79 ^A^	4.30 ± 0.08 ^C^	720.82 ± 37.04 ^A^	47.73 ± 0.66 ^AB^	3.30 ± 0.25 ^C^	139.97 ± 10.17 ^B^	28.49 ± 3.57 ^AB^	61.94 ± 9.34 ^A^
GK24	2.58 ± 0.44 ^E^	7.41 ± 0.96 ^B^	88.50 ± 10.64 ^D^	5.56 ± 1.16 ^CD^	3.62 ± 0.63 ^C^	44.43 ± 1.70 ^E^	26.09 ± 2.95 ^BC^	58.57 ± 5.85 ^AB^
GK25	10.58 ± 0.39 ^CD^	4.33 ± 0.49 ^C^	499.94 ± 23.93 ^B^	7.66 ± 0.92 ^CD^	3.30 ± 0.52 ^C^	149.77 ± 7.17 ^A^	32.62 ± 3.71 ^A^	53.86 ± 1.62 ^B^
GK31	23.04 ± 2.55 ^AB^	15.75 ± 4.22 ^A^	1.89 ± 0.68 ^I^	2.12 ± 1.44 ^D^	5.77 ± 0.12 ^C^	7.33 ± 1.98 ^GHI^	16.09 ± 0.64 ^EFG^	21.25 ± 0.05 ^E^
GK32	10.97 ± 1.18 ^CD^	2.70 ± 0.06 ^CDE^	178.97 ± 2.80 ^C^	52.43 ± 0.91 ^A^	206.95 ± 1.53 ^A^	117.60 ± 5.10 ^C^	16.50 ± 1.07 ^EFG^	29.54 ± 6.64 ^D^
GK33	ND ^E^	3.00 ± 1.60 ^CDE^	63.20 ± 0.40 ^E^	48.60 ± 4.60 ^AB^	232.10 ± 11.90 ^A^	107.00 ± 5.40 ^D^	18.60 ± 3.20 ^DE^	ND ^F^
GK41	5.13 ± 0.23 ^DE^	ND ^E^	25.65 ± 2.24 ^GH^	6.67 ± 0.75 ^CD^	41.13 ± 3.92 ^BC^	18.41 ± 0.43 ^F^	18.25 ± 1.61 ^DEF^	40.63 ± 3.20 ^C^
GK42	7.14 ± 0.38 ^DE^	3.18 ± 2.18 ^CD^	52.86 ± 9.96 ^EF^	35.74 ± 0.45 ^B^	88.21 ± 2.60 ^B^	1.28 ± 0.14 ^I^	23.57 ± 2.04 ^BCD^	56.22 ± 2.06 ^AB^
GK51	10.56 ± 1.84 ^CD^	2.69 ± 0.62 ^CDE^	16.99 ± 1.17 ^GHI^	9.57 ± 0.61 ^CD^	49.00 ± 4.50 ^BC^	13.01 ± 2.99 ^FG^	22.58 ± 5.56 ^CD^	31.97 ± 3.26 ^D^
GK52	6.64 ± 0.35 ^DE^	1.34 ± 0.46 ^DE^	34.44 ± 1.16 ^FG^	15.73 ± 1.00 ^C^	45.15 ± 2.29 ^BC^	2.75 ± 0.38 ^HI^	22.95 ± 1.31 ^BCD^	28.11 ± 4.09 ^DE^
Average	11.22 ± 8.23	3.44 ± 4.30	134.96 ± 220.53	20.5 ± 18.52	58.44 ± 75.77	76.15 ± 65.91	20.31 ± 6.35	31.30 ± 22.35

^1^ PK: *Pa* kimchi, GK: *Gat* kimchi; ^2^ TRP: tryptamine, PHE: β-phenylethylamine, PUT: putrescine, CAD: cadaverine, HIS: histamine, TYR: tyramine, SPD: spermidine, SPM: spermine; ^3^ mean values (±standard deviation) in the same column that are followed by different letters (A–I) are significantly different (*p* <0.05); ^4^ ND: not detected (<0.1 mg/kg).

**Table 3:** BA produced in assay media by lactic acid bacteria (LAB) strains isolated from *Pa* (green onion) kimchi and *Gat* (mustard leaf) kimchi samples.

Samples	Isolates	N ^1^	BA Production (μg/mL) ^2^			
			PHE	PUT	CAD	TYR
*Pa* kimchi	*Lactobacillus brevis*	5	1.67 ± 0.46 (1.27–2.39) ^3^	ND ^4^	ND	293.27 ± 9.11 (278.57–301.52)
		8	ND	317.07 ± 4.47 (314.13–322.21)	48.43 ± 4.47 (41.69–53.06)	ND
		1	0.98 ^5^	362.44	54.79	190.50
	*Lactobacillus sakei*	2	1.92 ± 1.18 (1.00–3.96)	ND	ND	122.67 ± 12.29 (113.98–131.36)
*Gat* kimchi	*Enterococcus faecium*	2	3.70 ± 0.26 (3.51–3.88)	ND	ND	264.34 ± 7.40 (259.10–269.57)
	*Lactobacillus brevis*	5	1.55 ± 0.53 (1.47–2.34)	ND	ND	338.51 ± 25.65 (300.61–365.96)
		2	ND	318.05 ± 4.94 (313.43–320.42)	42.99 ± 6.71 (39.24–47.73)	ND
	*Leuconostoc mesenteroides*	2	1.69 ± 0.31 (1.47–1.91)	ND	ND	122.67 ± 12.29(145.14–301.67)

^1^ N: the number of strains tested; ^2^ PHE: β-phenylethylamine, PUT: putrescine, CAD: cadaverine, TYR: tyramine (tryptamine, histamine, spermidine, and spermine were all not detected); ^3^ mean ± standard deviation (the range from minimum to maximum); ^4^ ND, not detected (<0.1 μg/mL); ^5^ mean value obtained from a single strain.
